# Mood and quality of life changes in pregnancy and postpartum and the effect of a behavioral intervention targeting excess gestational weight gain in women with overweight and obesity: a parallel-arm randomized controlled pilot trial

**DOI:** 10.1186/s12884-019-2196-8

**Published:** 2019-01-29

**Authors:** Abby D. Altazan, Leanne M. Redman, Jeffrey H. Burton, Robbie A. Beyl, Loren E. Cain, Elizabeth F. Sutton, Corby K. Martin

**Affiliations:** Pennington Biomedical Research Center, Louisiana State University, 6400 Perkins Road, Baton Rouge, LA 70808 USA

**Keywords:** Pregnancy, Mental health, Quality of life, Gestational weight gain

## Abstract

**Background:**

Intensive lifestyle interventions in pregnancy have shown success in limiting gestational weight gain, but the effects on mood and quality of life in pregnancy and postpartum are less known. The purpose was to quantify changes in mental and physical quality of life and depressive symptoms across pregnancy and the postpartum period, to determine the association between gestational weight gain and change in mood and quality of life, and to assess the effect of a behavioral intervention targeting excess gestational weight gain on these outcomes.

**Methods:**

A three group parallel-arm randomized controlled pilot trial of 54 pregnant women who were overweight or obese was conducted to test whether the SmartMoms® intervention decreased the proportion of women with excess gestational weight gain. Individuals randomized to Usual Care (*n* = 17) did not receive any weight management services from interventionists. Individuals randomized to the SmartMoms® intervention (*n* = 37) were provided with behavioral weight management counseling by interventionists either in clinic (In-Person, *n* = 18) or remotely through a smartphone application (Phone, *n* = 19). In a subset of 43 women, mood and mental and physical quality of life were assessed with the Beck Depression Inventory-II and the Rand 12-Item short form, respectively, in early pregnancy, late pregnancy, 1–2 months postpartum, and 12 months postpartum.

**Results:**

The SmartMoms® intervention and Usual Care groups had higher depressive symptoms (*p* < 0.03 for SmartMoms® intervention, *p* < 0.01 for Usual Care) and decreased physical health (*p* < 0.01) from early to late pregnancy. Both groups returned to early pregnancy mood and physical quality of life postpartum. Mental health did not change from early to late pregnancy (*p* = 0.8), from early pregnancy to 1–2 months (*p* = 0.5), or from early pregnancy to 12 months postpartum (*p* = 0.9), respectively. There were no significant intervention effects. Higher gestational weight gain was associated with worsened mood and lower physical quality of life across pregnancy.

**Conclusion:**

High depressive symptoms and poor quality of life may be interrelated with the incidence of excess gestational weight gain. The behavioral gestational weight gain intervention did not significantly impact these outcomes, but mood and quality of life should be considered within future interventions and clinical practice to effectively limit excess gestational weight gain.

**Trial registration:**

NCT01610752, Expecting Success, Registered 31 May 2012.

**Electronic supplementary material:**

The online version of this article (10.1186/s12884-019-2196-8) contains supplementary material, which is available to authorized users.

## Background

More than two-thirds of pregnant women exceed the Institute of Medicine (IOM) 2009 gestational weight gain (GWG) recommendations [1–3]. Excess gestational weight gain is problematic for women entering pregnancy at any size [4], and pregnant women with overweight and obesity are at increased risk for adverse maternal outcomes including gestational diabetes, hypertension [5, 6], Cesarean delivery [6, 7], and postpartum weight retention [2, 8], and infant outcomes including large for gestational age at birth [9] and obesity in childhood [10, 11]. Limiting gestational weight gain as recommended by the IOM guidelines may, therefore, have beneficial effects for both mother and infant at delivery and later in life highlighting the critical need for interventions promoting appropriate weight gain during pregnancy [12].

In recent years, many diet, physical activity, and combined interventions have been implemented in pregnancy and their effects on gestational weight gain and outcomes have been studied. A meta-analysis recently showed an overall benefit of lifestyle interventions for limiting gestational weight gain compared to standard of care, but did not indicate reduced risk for adverse maternal and infant outcomes [13]. Similar to previous research, the Lifestyle Interventions for Expectant Moms (LIFE-Moms) Consortium, which included various interventions aimed to reduce excess gestational weight gain at seven clinical centers [14], found that diet and physical activity interventions significantly reduced gestational weight gain with no significant impact on pre-eclampsia, gestational diabetes, Cesarean delivery, or infant birth weight [15]. One of the interventions tested as part of the LIFE-Moms Consortium was named SmartMoms®. The SmartMoms® intervention was grounded on objectively measuring body weight and steps and providing data-driven feedback to participants [16–20]. Participants in the SmartMoms® intervention received a personalized IOM 2009 gestational weight gain graph and dietary intake and physical activity counseling to encourage and assist with adherence to the IOM 2009 gestational weight gain guidelines [1, 21]. The SmartMoms® intervention was delivered in a traditional clinic based setting (In-Person) or remotely through a smartphone application (Phone) in the Expecting Success pilot trial, and the primary outcome results have been reported previously [1]. Briefly, a significantly smaller proportion of women in the SmartMoms® intervention exceeded the IOM 2009 gestational weight gain guidelines (In-Person: 55.6%, 10/18 and Phone: 57.9%, 11/19) compared to Usual Care (84.6%, 11/13) (*p* < 0.02). Weight gain was equivalent in the SmartMoms® In-Person and SmartMoms® Phone groups, and the SmartMoms® intervention (In-Person and Phone combined) was effective in reducing overall gestational weight gain as compared to the Usual Care group (Usual Care: LS mean 12.8, SE 1.5 kg and SmartMoms® intervention: LS mean 9.2 SE 0.9 kg; *p* = 0.04).

While the physiological changes that occur in pregnant women in response to lifestyle interventions are well described, the effect of lifestyle modifications on mood, general well-being, and quality of life is less known [22]. Pregnancy may cause women to encounter new stressors, and, therefore, the potential for anxiety, depression, and emotional eating behaviors may increase [2, 12, 23]. In a focus group, low income pregnant women with overweight and obesity reported tiredness, lack of motivation, and family support as barriers to physical activity in pregnancy [12]. Another study with structured interviews reported that pregnant women used eating as a coping mechanism to relieve emotional and physical discomfort [2], and physiological symptoms such as nausea and vomiting had a significant effect on health related quality of life [24]. Furthermore, the physical, hormonal, and behavioral changes of pregnancy may also be related to changes in perceived physical quality of life [25]. Quality of life scores are lower in pregnant women compared to women in the general U.S. population [24]. Health related quality of life decreases across pregnancy [24–26], and the incidence of depressive symptoms has been shown to increase across pregnancy [25]. Nonetheless, changes in maternal mood and quality of life have been seldom studied in conjunction with lifestyle intervention studies in pregnant women [23], and to our knowledge, no study has 1) evaluated change in mood and quality of life throughout pregnancy and to 12 months postpartum, 2) examined the association between gestational weight gain and change in mood and quality of life, or 3) determined if an intervention that successfully limited excessive gestational weight gain affects change in mood and quality of life. Hence, the purpose of this investigation was: 1) to quantify changes in mental and physical quality of life and depressive symptoms across pregnancy and the postpartum period, 2) to determine if gestational weight gain was associated with changes in mood and quality of life, and 3) to assess the effect of a behavioral intervention targeting excessive gestational weight gain on mood and quality of life.

## Methods

### Study design and intervention

The Expecting Success (NCT01610752) was a three group parallel-arm randomized controlled pilot trial conducted in Baton Rouge, Louisiana, United States. The purpose was to implement a personalized weight management program for women entering pregnancy with overweight and obesity to improve adherence to the 2009 IOM guidelines for appropriate gestational weight gain [1]. Clinical outcomes were evaluated across pregnancy, and mothers and infants were followed until 12 months postpartum. Eligible participants were randomized by an unblinded staff member before 13 weeks 5 days gestation to one of three groups: 1) No intervention (Usual Care), 2) SmartMoms® intervention in-person (In-Person), or 3) SmartMoms® intervention via smartphone (Phone). Random assignment was stratified by enrollment body mass index (BMI) class and was prepared prior to study initiation by the biostatistician who numbered and sealed envelopes until an unblinded staff member could retrieve the appropriate envelope.

Individuals randomized to Usual Care were under the usual care of their obstetrician and did not receive any weight management services from interventionists. Individuals randomized to the SmartMoms® intervention were provided with behavioral weight management counseling by interventionists either in a clinic based setting (In-Person) or remotely through a smartphone application (Phone). The SmartMoms® intervention consisted of 18 lessons with diet and behavior modification strategies weekly between 13 and 24 weeks gestation and every-other-week from 25 weeks gestation to delivery (Additional file 1). Individuals randomized to the SmartMoms® intervention received a personalized IOM 2009 gestational weight gain graph, a wireless internet-connected bathroom scale, and a pedometer. Individuals in the SmartMoms® In-Person group interacted with interventionists in face-to-face sessions and tracked their weight and steps by hand in sessions. Individuals in the SmartMoms® Phone group interacted with the interventionists at least once per week through the smartphone application and email, text, or phone contact, and weights and step counts were wirelessly transmitted to the application and plotted into graphs within the application so that individuals could track their progress in near real-time [1].

### Participants

Pregnant women were recruited through advertisements and targeted emails and referrals from local obstetricians [27]. Eligibility criteria included pregnant women with overweight or obesity aged 18 to 40 years who were: carrying viable singletons, English speaking, and medically cleared by their obstetricians and the study medical investigator with no history or current psychotic disorder or major depressive episodes. Gestational age was calculated using the self-reported last menstrual period date if the dating ultrasound confirmed the last menstrual period date within 7 days or using the dating ultrasound if the last menstrual period date was not confirmed. Eligible pregnant women were enrolled between February 2013 to May 2014, and the study was completed in October 2015. The study was approved and monitored by the Pennington Biomedical Research Center and Woman’s Hospital Institutional Review Boards and was registered as a clinical trial (NCT01610752). CONSORT guidelines were followed for this study. Written informed consent was obtained for all participants prior to the initiation of procedures, and financial compensation and small incentive items were provided to offset transportation costs and for participants’ time.

### Clinical assessments

Clinical assessments were performed by staff members who were blinded to the group assignment. Body weight was measured to the nearest 0.1 kg on a calibrated scale (GSE, Livonia, Michigan, United States) after an overnight fast while wearing only a pre-weighed hospital gown and undergarments. At screening, height was measured twice using a wall-mounted stadiometer with head in the Frankfort plane. Enrollment BMI was determined using the first measured clinic weight and height at screening before 13 weeks 5 days gestation, and eligible participants had enrollment BMI between 25.0 and 39.9 kg/m^2^. Overall gestational weight gain was calculated as the difference between the measured clinic weight between 35 weeks and 36 weeks 6 days gestation and the first measured clinic weight before 13 weeks 5 days gestation. Gestational weight gain per week was used to calculate the proportion of women based on enrollment BMI with excessive GWG per the IOM guidelines. Sociodemographic data including age, race, parity, total household income, and education were obtained from self-report questionnaires.

### Psychological assessments

Self-report questionnaires were completed at screening (before 13 weeks 5 days gestation), 35 to 36 weeks 6 days gestation, 1 to 2 months postpartum, and 12 months postpartum. Questionnaires were provided by staff members who were blinded to the group assignment, and participants completed paper-based questionnaires by themselves in private. Mood and depressive symptoms were assessed with the Beck Depression Inventory II (BDI-II) with scores ranging from 0 to 63, where higher scores indicate worse mood and more severe symptoms of depression [28]. Quality of life was measured with the Rand 12-Item Short Form (SF-12) [29]. The SF-12 includes a mental health composite score (MCS) consisting of 6 items assessing mental health, role limitations due to emotional problems, social functioning, and vitality and a physical health composite score (PCS) consisting of 6 items assessing physical functioning, role limitations due to physical health problems, bodily pain, and general health. The SF-12 mental and physical composite scores range from 0 to 100 and are compared to age-specific national norm data with a mean score of 50.0 and a standard deviation of 10.0, where higher scores indicate better quality of life.

### Statistical analysis

As noted elsewhere [1] and herein, the SmartMoms® In-Person and Phone intervention groups were successful in attenuating gestational weight gain as compared to the Usual Care group and overall gestational weight gain was shown to be equivalent between the SmartMoms® In-Person and Phone intervention groups (*p* = 0.04 equivalence). Therefore, they were combined into a single SmartMoms® intervention group to decrease the type I error rate and provide a better variance estimate.

The study was conducted as a pilot and feasibility study to follow 54 mother-infant dyads. The outcomes presented here were secondary measures and not part of the a-priori power analysis. The effect sizes reported herein are small according to Cohen’s effect size criteria [30] with almost all (10 of 12) effect sizes being 0.20 or smaller. Confidence in the conclusions from the analyses was further supported by post-hoc power calculations, which demonstrated that the observed effect sizes were sufficiently small to necessitate very large sample sizes ranging from 768 to 250,000 participants to detect significant between group differences with power equal to 0.80, alpha equal to 0.05, and two-sided tests. Thus, we recognize that the study had a relatively small sample size, though a larger sample size would not have affected the study’s conclusions.

Socioeconomic status was examined using two composite measures: socioeconomic composite factor score and poverty to income ratio. Socioeconomic composite factor score is a standardized z score that incorporates income and education and controls for race with higher values indicating higher socioeconomic status. Poverty to income ratio is the ratio of an individual’s income to poverty threshold based on family size. Baseline comparisons between the combined SmartMoms® intervention and the Usual Care groups used ANOVA for continuous variables and Chi-squared tests for categorical variables. The repeated measures over time were estimated using a mixed effect linear model. The covariance between the repeated measures over time was accounted for using an unstructured covariance matrix. Effect sizes were calculated from the mean difference between the Usual Care and SmartMoms® intervention groups over the standard deviation difference between the groups. Associations between the continuous predictors (BDI-II, SF-12) and overall gestational weight gain were estimated using regression beta coefficients and tested for changes from early pregnancy to each of the additional time points using t-tests. Statistical analyses were completed using SAS/STAT® software, Version 9.4 of the SAS System for Windows (Cary, NC, USA). All tests were performed with significance level α = 0.05.

## Results

### Participant characteristics

Fifty-four women were enrolled in the study with 37 women in the SmartMoms® intervention and 17 women in the Usual Care group. Participants who had psychological assessment data at baseline prior to randomization (13 weeks, 5 days) and at least one follow up visit (*n* = 43) were included in this analysis. Four women dropped due to miscarriage and 7 women did not complete psychological assessments after baseline so 11 women were not included. Of the 11 women, 5 were dropped from the SmartMoms® intervention, and 6 were dropped from the Usual Care group. The demographic characteristics of the SmartMoms® Intervention and Usual Care groups were similar with no statistical differences observed between groups at enrollment (Table 1). The majority of participants were White and nulli- or primiparous with a mean age of 29.2 years and mean weight of 83.3 kg at enrollment.

**Table 1:** Baseline Characteristics by Treatment Group

Characteristic	Usual Care (*n* = 17)	SmartMoms® Intervention (*n* = 37)	All (*n* = 54)	p between groups
Gestational age, wks	9.6 ± 1.0	10.2 ± 1.2	10.0 ± 1.2	0.06
Age, y	29.5 ± 5.1	29.1 ± 4.4	29.2 ± 4.6	0.78
Race				0.25
Black	6 (35.3%)	7 (18.9%)	13 (24.1%)	
White	11 (64.7%)	27 (73.0%)	38 (70.4%)	
Other	0 (0%)	3 (8.1%)	3 (5.6%)	
Parity	0.6 ± 0.5	0.8 ± 1.0	0.8 ± 0.8	0.34
Weight, kg	86.2 ± 12.1	82.0 ± 12.0	83.3 ± 12.1	0.24
Enrollment BMI	31.1 ± 3.7	31.0 ± 4.2	31.0 ± 4.0	0.96
Enrollment BMI Group				0.51
Overweight, 25.0–29.9 kg/m^2^	9 (52.9%)	16 (43.24%)	25 (46.3%)	
Obese, 30.0–40.0 kg/m^2^	8 (47.1%)	21 (56.8%)	29 (53.7%)	
Total Household Income, per year				0.92
< $5000–$39,999	7 (41.2%)	16 (43.2%)	23 (42.6%)	
$40,000–$99,999	5 (29.4%)	12 (32.4%)	17 (31.5%)	
$100,000 and above	5 (29.4%)	9 (24.3%)	14 (25.9%)	
Education				0.26
Some high school	0 (0%)	1 (2.7%)	1 (1.9%)	
High school diploma/GED/1-3y college, business or technical school	7 (41.2%)	11 (29.7%)	18 (33.3%)	
College degree	4 (23.5%)	18 (48.7%)	22 (40.7%)	
Post graduate education	6 (35.3%)	7 (18.9%)	13 (24.1%)	
Socioeconomic Composite Factor	0.1 ± 1.0	−0.1 ± 1.0	0 ± 1.0	0.47
Poverty to Income Ratio	3.9 ± 2.8	3.5 ± 2.8	3.6 ± 2.8	0.70

Continuous values are displayed as mean ± SD and categorical variables as frequency (%). Socioeconomic composite factor is a standardized z score that incorporates income and education and controls for race. *P* values were derived from ANOVA for continuous variables and Chi-squared test for categorical variables

### Gestational weight gain

Similarly to the primary outcome results described previously [1], in the subset of 43 individuals included in this analysis, the proportion of women who had gestational weight gain that exceeded the IOM 2009 gestational weight gain guidelines was 56.3% (18/32) in the SmartMoms® intervention and 81.8% (9/11) in the Usual Care group (*p* = 0.17). Women in the SmartMoms® intervention had less overall gestational weight gain as compared to the women in the Usual Care group (Usual Care: LS mean 12.8, SE 1.5 kg and SmartMoms® intervention: LS mean 8.7 SE 0.9 kg; *p* = 0.03).

### Mood

**Fig. 1:**
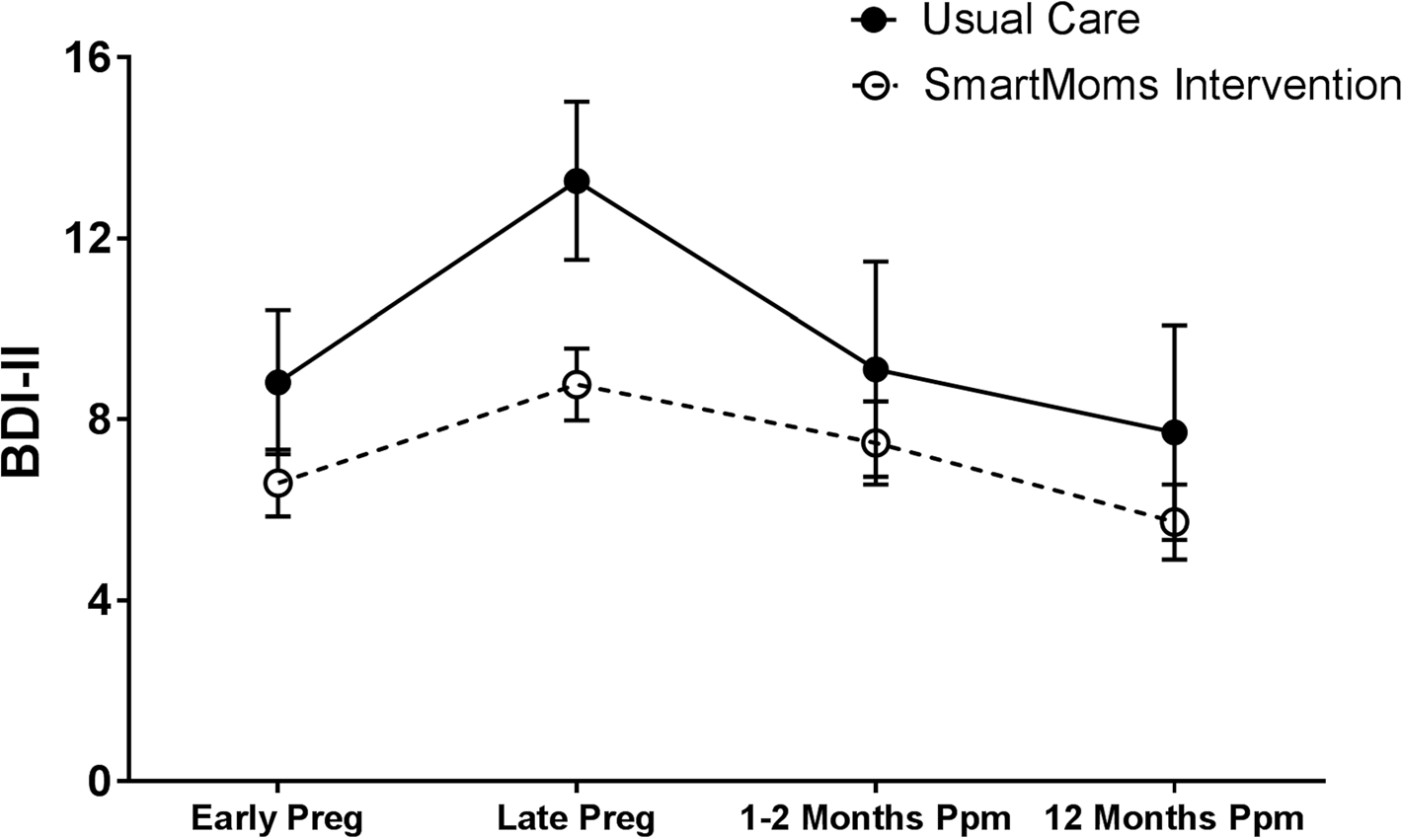
Change in Mood (BDI-II) Over Time for the SmartMoms® Intervention (open circles with dashed lines) and Usual Care (closed circles with solid lines) groups. Data is displayed as Mean and SE and was derived using repeated measures over time in a mixed effect linear model. BDI-II: Beck Depression Inventory II, Preg: Pregnancy, Ppm: Postpartum, SE: Standard error

There was not a significant group main effect (*p* = 0.74), but there was a significant time main effect (*p* < 0.0001) and a group x time interaction (*p* = 0.04) for BDI-II scores. Maternal depressive symptoms worsened (BDI-II scores increased) significantly over time from early pregnancy to late pregnancy in both the SmartMoms® intervention (*p* < 0.01) and Usual Care (*p* = 0.03) groups (Fig. 1 and Table 2). At 1 to 2 months and 12 months postpartum, BDI-II scores returned to early pregnancy levels in both groups (Fig. 1). Individual post-hoc tests that examined the interaction found no significant differences in change in mood at any time point by group, and Fig. 1 and Table 2 suggest that the group x time interaction was driven by increased depression scores in the Usual Care group from early to late pregnancy (Fig. 1 and Table 2).

**Table 2:** Early pregnancy values and estimated change in mood and quality of life

		Usual Care (*n* = 11)		SmartMoms® Intervention (*n* = 32)		Between Group		
		Mean (SE)	p	Mean (SE)	p	Difference (SE)	p	ES
Mood								
BDI-II	Early Pregnancy	8.82 (1.35)		6.59 (0.79)		−2.22 (1.56)	0.16	0.22
	Δ Late Pregnancy	4.45 (1.62)	**< 0.01**	2.06 (0.96)	**0.03**	−2.40 (1.88)	0.21	0.20
	Δ 1–2 months ppm	−0.66 (1.74)	0.71	0.86 (0.96)	0.37	1.52 (1.99)	0.45	0.12
	Δ 12 months ppm	−2.98 (1.90)	0.12	−0.51 (0.97)	0.12	2.47 (2.14)	0.25	0.18
Quality of Life								
SF-12 MCS	Early Pregnancy	51.13 (1.41)		50.53 (0.83)		−0.6 (1.63)	0.72	0.06
	Δ Late Pregnancy	−0.80 (2.60)	0.76	1.49 (1.54)	0.34	2.28 (3.02)	0.45	0.12
	Δ 1–2 months ppm	−1.97 (2.81)	0.49	−1.7 (1.54)	0.27	0.27 (3.21)	0.93	0.01
	Δ 12 months ppm	−0.37 (3.08)	0.9	−1.83 (1.56)	0.24	−1.46 (3.45)	0.67	0.07
SF-12 PCS	Early Pregnancy	49.37 (1.65)		51.14 (0.97)		1.76 (1.91)	0.36	0.14
	Δ Late Pregnancy	−6.99 (1.99)	**< 0.01**	−8.32 (1.18)	**< 0.01**	−1.33 (2.31)	0.57	0.09
	Δ 1–2 months ppm	4.20 (2.19)	0.06	0.41 (1.18)	0.73	−3.78 (2.49)	0.13	0.23
	Δ 12 months ppm	4.65 (2.46)	0.06	3.37 (1.20)	**< 0.01**	−1.28 (2.74)	0.64	0.07

Continuous values are displayed as mean (SE) and effect size is reported using group differences (mean/SD), and p values were derived using repeated measures over time in a mixed effect linear model. Significance was assumed when *p* < 0.05, as shown in bold

### Quality of life

**Fig. 2:**
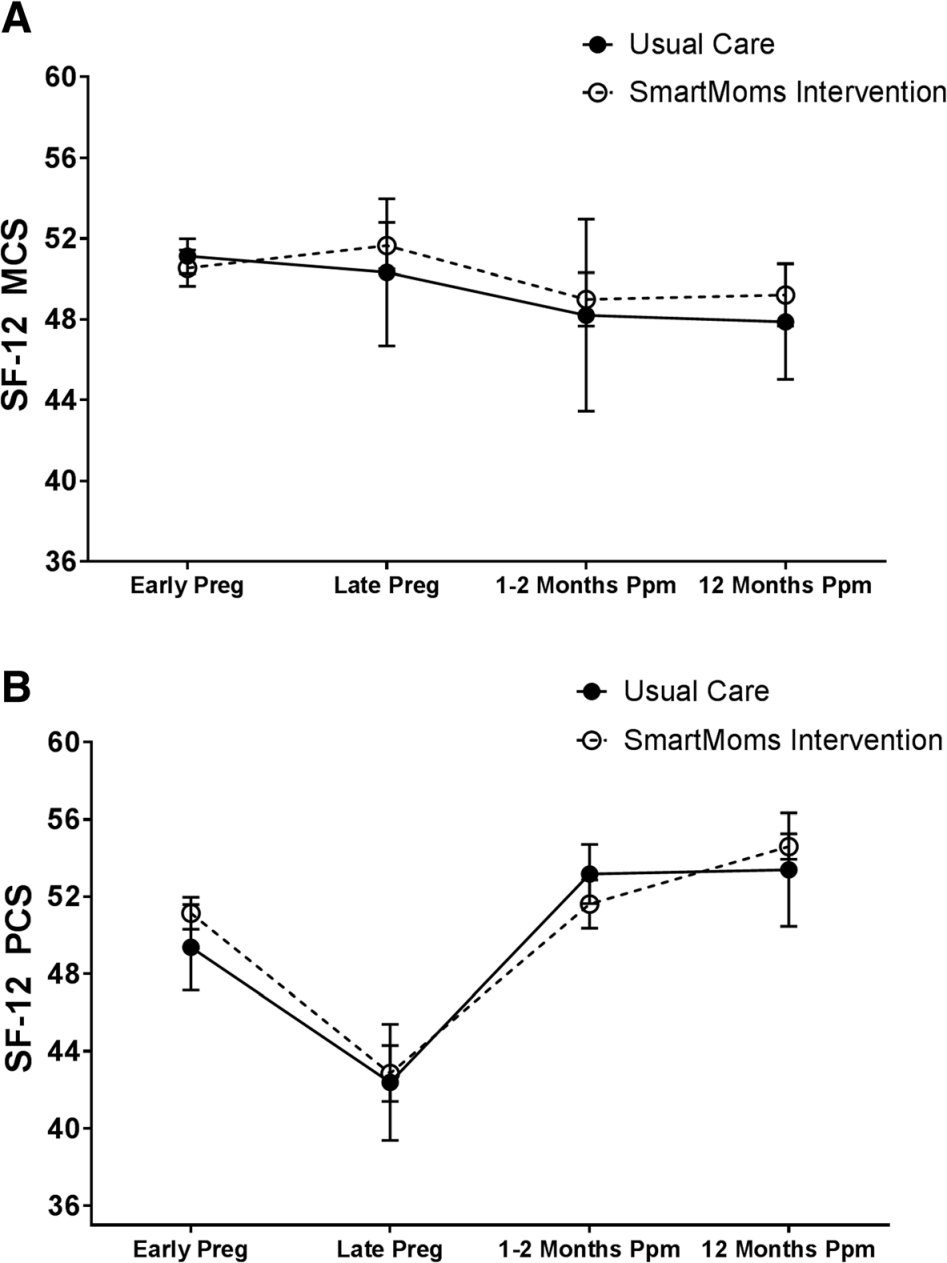
Mental Health (SF-12 MCS) (Panel **a**) and Physical Health (SF-12 PCS) (Panel **b**) Over Time for the SmartMoms® Intervention (open circles with dashed lines) and Usual Care (closed circles with solid lines) groups. Data is displayed as Mean and SE and was derived using repeated measures over time in a mixed effect linear model. SF-12: Rand 12-Item Short Form, MCS: Mental health composite score, PCS: Physical health composite score, Preg: Pregnancy, Ppm: Postpartum, SE: Standard error

There was no time main effect (*p* = 0.39), no group main effect (*p* = 0.89), and no group x time interaction (*p* = 0.55) for SF-12 mental health composite scores (Fig. 2 Panel A, Table 2). There was an overall time effect on SF-12 physical health composite score (*p* < 0.0001) with no group main effect (*p* = 0.24) or group x time interaction (*p* = 0.64). As shown in Fig. 2 Panel B and Table 2, physical health aspects of quality of life significantly decreased in both the SmartMoms® intervention and the Usual Care groups from early to late pregnancy (*p* < 0.01 for both), then increased to early pregnancy or slightly above early pregnancy values at 1 to 2 months postpartum and 12 months postpartum.

### Correlations between GWG and questionnaire change scores

**Fig. 3:**
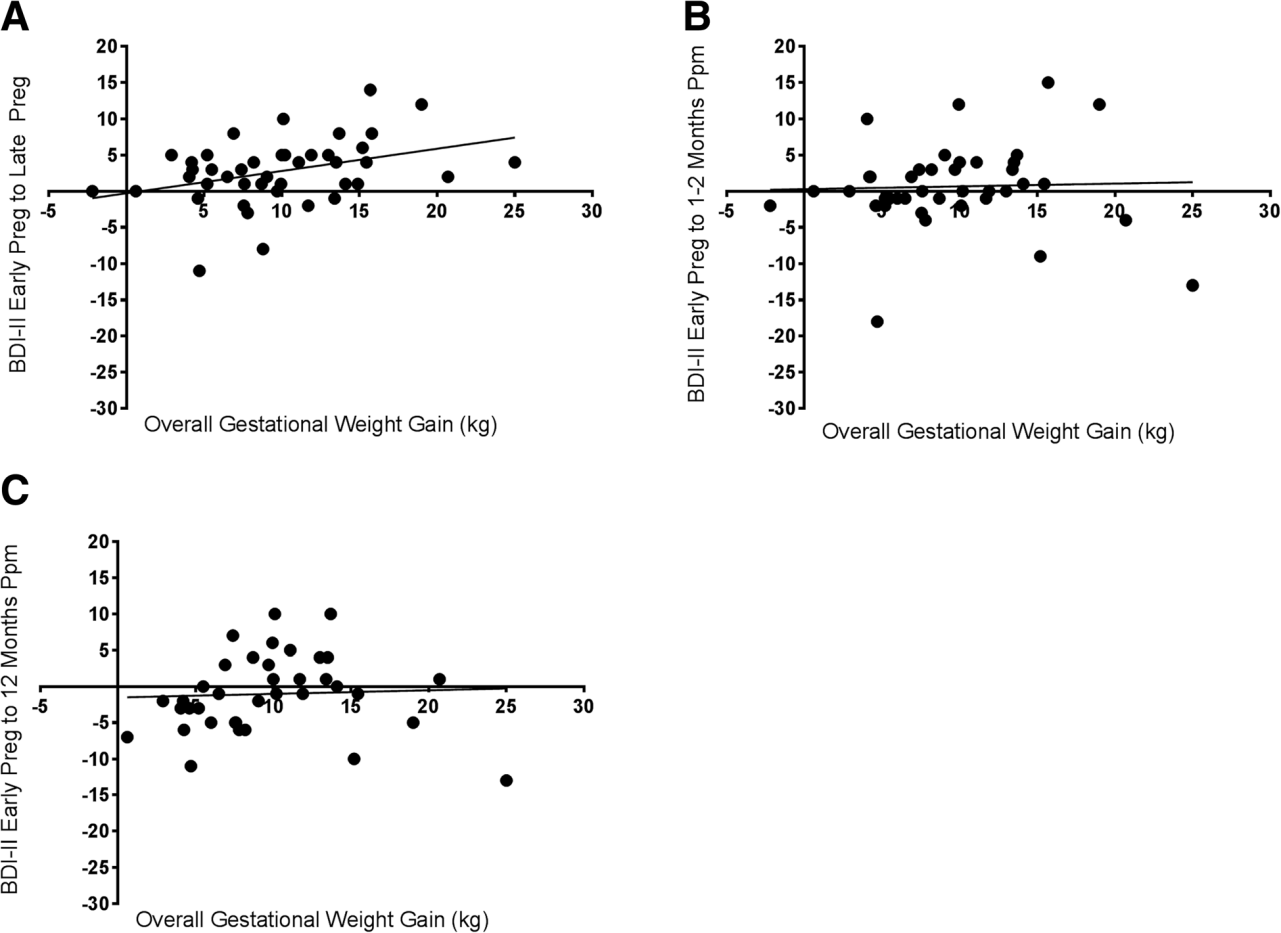
Association between Overall Gestational Weight Gain and Change in Mood (BDI-II) from early pregnancy to late pregnancy (Panel **a**), early pregnancy to 1–2 months postpartum (Panel **b**), and early pregnancy to 12 months postpartum (Panel **c**). Data is presented as individual overall gestational weight gain versus change in BDI-II scores and was derived using repeated measures over time in a mixed effect linear model. BDI-II: Beck Depression Inventory II, Preg: Pregnancy, Ppm: Postpartum, kg: kilogram

A positive association was observed between overall gestational weight gain and the difference in mood scores assessed by the BDI-II from early to late pregnancy (*p* < 0.0001) (Fig. 3Panel A). There was no significant association between overall gestational weight gain and change in mood assessed by the BDI-II from early pregnancy to 1 to 2 and 12 months postpartum (Fig. 3Panel B and Panel C).

**Fig. 4:**
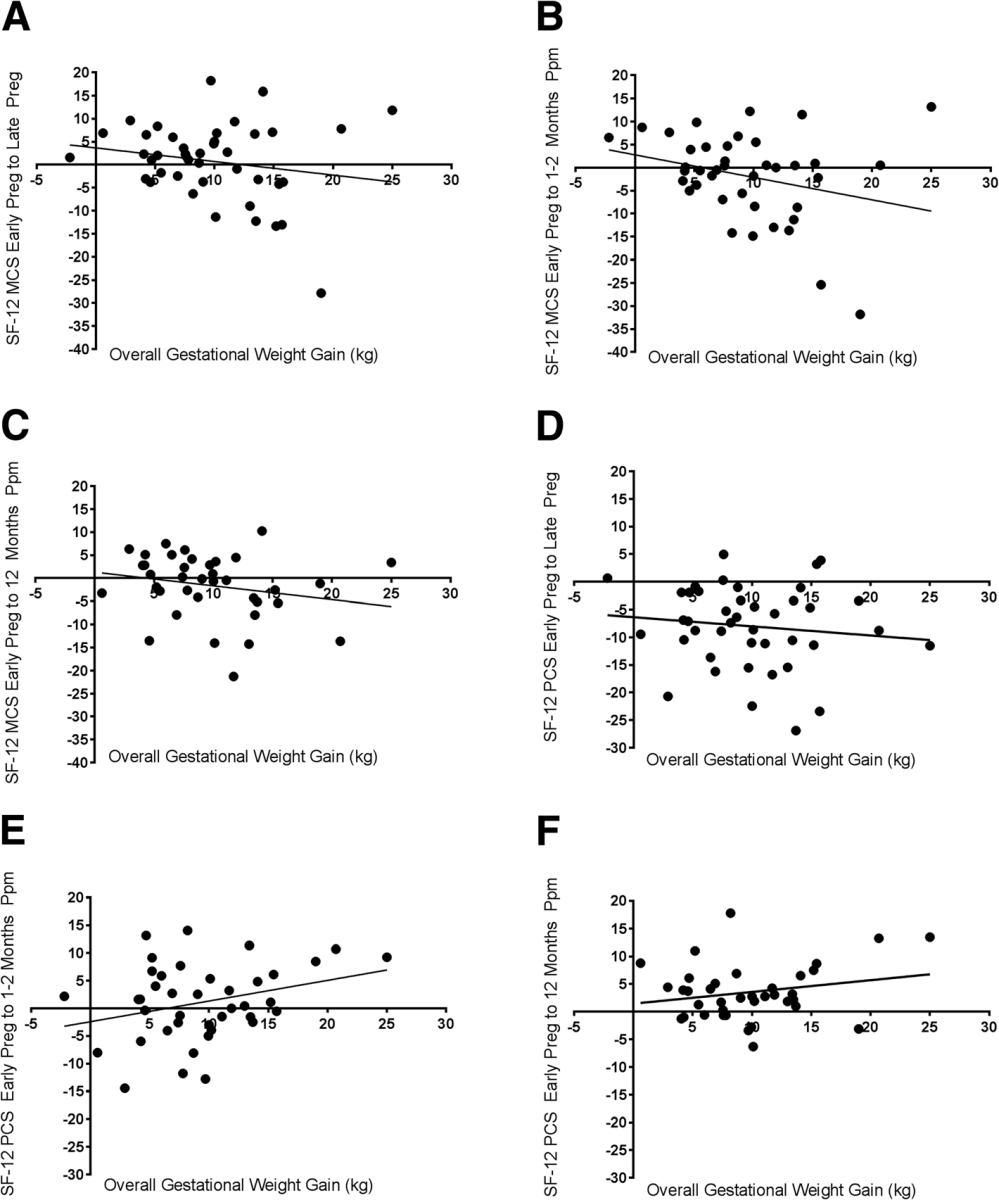
Association between Overall Gestational Weight Gain and Change in Mental Health (SF-12 MCS) from early pregnancy to late pregnancy (Panel **a**), early pregnancy to 1–2 months postpartum (Panel **b**), and early pregnancy to 12 months postpartum (Panel **c**) and Physical Health (SF-12 PCS) from early pregnancy to late pregnancy (Panel **d**), early pregnancy to 1–2 months postpartum (Panel **e**), and early pregnancy to 12 months postpartum (Panel **f**). Data is presented as individual overall gestational weight gain versus change in SF-12 scores and was derived using repeated measures over time in a mixed effect linear model. MCS: Mental health composite score, PCS: Physical health composite score, Preg: Pregnancy, Ppm: Postpartum, kg: kilogram

There was no association between the change in mental health composite scores from early to late pregnancy and overall gestational weight gain (Fig. 4 Panel A). There was a negative association between overall gestational weight gain and the change in physical health composite scores from early to late pregnancy (*p* = 0.0042) (Fig. 4 Panel D). In addition, at 12 months postpartum, there was a negative association between overall gestational weight gain and the difference in mental health composite scores (*p* = 0.0226) (Fig. 4 Panel C) and a positive association between overall gestational weight gain and the difference in physical health composite scores (*p* = 0.0078) (Fig. 4 Panel F).

### Correlations between demographics and questionnaire change scores

Neither maternal age nor parity was associated with physical or mental quality of life or mood in early pregnancy or changes from early to late pregnancy. In addition, socioeconomic composite factor was not correlated with physical or mental quality of life or mood in early pregnancy or changes from early to late pregnancy. There were no observed associations between poverty to income ratio and physical or mental quality of life or mood in early pregnancy or changes from early to late pregnancy (Table 3).

**Table 3:** Correlations between demographic characteristics and questionnaire change scores

	Age	Parity	Socioeconomic Composite Factor	Poverty to Income Ratio
	r	r	r	r
1st Trimester BDI-II	−0.05	0.08	−0.23	−0.03
Δ 3rd Trimester BDI-II	0.01	0.02	0.01	0.22
1st Trimester SF-12 MCS	−0.07	0.16	0.04	−0.20
Δ 3rd Trimester SF-12 MCS	−0.20	−0.11	< 0.01	− 0.21
1st Trimester SF-12 PCS	0.04	0.02	0.23	0.12
Δ 3rd Trimester SF-12 PCS	0.01	0.07	0.20	−0.04

Pearson’s correlation analysis was used to generate r and *p* values

## Discussion

The objectives of this investigation were to describe the natural changes in maternal mood and depressive symptoms and mental and physical quality of life across pregnancy and the postpartum period and to examine the effect of the behavioral intervention on these factors. Participants in both the SmartMoms® intervention and Usual Care groups had significantly higher depressive symptoms from early to late pregnancy. Compared to the Usual Care group, the SmartMoms® intervention participants had smaller increases in depressive symptoms during the study period, and this effect appears to be driven by the Usual Care group’s increase in depressive symptoms from early to late pregnancy. Consistent with previous studies [25, 26], mental health aspects of quality of life did not vary over pregnancy or the postpartum period. Change in physical health aspects of quality of life varied over time, with physical quality of life decreasing from early to late pregnancy, which has been previously reported [25, 26]. Our study further demonstrated that physical quality of life rebounds quickly by 1 to 2 months postpartum and remains at early pregnancy levels or slightly above until at least 12 months postpartum.

Other novel findings from the current study were that higher overall gestational weight gain was associated with worsened mood and lower physical quality of life from early to late pregnancy, and higher gestational weight gain was associated with decreased mental quality of life from early pregnancy to 12 months postpartum. These results are novel and in the expected direction. However, the study also found that higher overall gestational weight gain was associated with improved physical quality of life from early pregnancy to 12 months postpartum. To our knowledge, this is an entirely new and unexpected finding that requires replication and further study. We did not observe any significant associations between demographic or sociodemographic factors on mood or quality of life in early pregnancy or changes in mood or quality of life from early to late pregnancy.

There are physiological changes that naturally occur early in pregnancy and extend into the postpartum period [23, 31] that may contribute to decreased mood and physical health in pregnancy. Weight gain during pregnancy can have a significant effect on mood and physical health as a result of gaining more weight than ever before, the physical change of the body’s center of gravity, and changes in mobility and gait [32]. Moreover, pregnant women often experience gastrointestinal issues such as nausea, vomiting, constipation, heartburn, and indigestion that may also contribute to decreases in quality of life. In preparation for childbirth, hormonal changes occur to loosen joints commonly leading to back pain and sciatica. Especially later in pregnancy, women experience poor sleep due to frequent bathroom trips, fetal movement, and aches and pains. It seems that regardless of maternal health status or pre-existing complications, worsening mood and decreases in physical quality of life occur as a result of these normal physiological changes. Interestingly, the mood and physical quality of life changes that we observed from early to late pregnancy did not persist in the postpartum period, suggesting that these effects are temporary and restricted to pregnancy.

An important finding from the study was that there were no negative intervention effects on mood or quality of life. This stability in mental health composite scores suggests no untoward mental effects from the gestational weight gain intervention during pregnancy. It is important that deployed lifestyle interventions are sufficiently intensive to promote behavior change, and this study confirmed that, similar to traditional weight loss or weight maintenance interventions, these behavior change strategies have no negative impact on mood or quality of life throughout pregnancy. Weight loss achieved from intensive lifestyle intervention has been shown to improve mood and quality of life in non-pregnant adults of all BMI classes [33–35]; however we did not observe any significant positive effect on mood and quality of life from an intervention aimed to limit gestational weight gain. Since the SmartMoms® intervention was aimed at appropriate weight gain as opposed to a traditional weight loss intervention, it is plausible that the improvements in mood and quality of life that have been shown in successful weight loss interventions may not exist, but may be tied directly to weight loss achieved rather than the behavior or lifestyle changes that are included in such interventions. In addition, a recent review concluded that future studies are needed to elucidate the relationship between weight loss interventions and mood and quality of life in the postpartum period [36], and our study indicates that affecting mood and quality of life postpartum requires an intervention to be delivered during that time and that the intervention must be powerful enough to improve mood and quality of life beyond early pregnancy levels.

A strength of this study is the detailed description on the course of quality of life and mood across pregnancy and until 12 months postpartum. In addition, this study is the first, to our knowledge, to describe a lifestyle intervention that successfully limited excessive gestational weight gain without negatively affecting quality of life or mood across pregnancy as evidenced by no treatment effects between early and late pregnancy assessments. The lack of negative effects of the lifestyle intervention on mood and quality of life indicate that interventions geared to limit energy intake and increase physical activity during pregnancy are safe. This study suggests that higher gestational weight gain may have negative effects on mood and physical quality of life during pregnancy. The association between gestational weight gain and change in physical quality of life from early pregnancy to 12 months postpartum is counterintuitive and requires further study. Including 12 month follow up data is another strength to show the postpartum effects of the intervention.

This pilot study is limited due to small sample size and was not powered for this secondary analysis. Nonetheless, this novel preliminary data provides valuable insight into mood and quality of life during pregnancy and postpartum period. Adequately powered studies should be conducted to assess the extent of lifestyle intervention effects on mood, depressive symptoms, and quality of life in pregnancy. Another limitation of this study was the use of self-report questionnaires to assess mood and quality of life although mood and quality of life are traditionally measured by the questionnaires used in this study. Including only women with overweight and obesity prior to pregnancy may be a limitation since quality of life and mood has been previously correlated to BMI [37, 38].

## Conclusions

In this study, women with overweight and obesity prior to pregnancy experienced decreased mood and physical health from early to late pregnancy and returned to early pregnancy mood and quality of life in the postpartum period regardless of inclusion in the behavioral intervention aimed at limiting gestational weight gain. Both mood and physical quality of life returned to near or slightly better than early pregnancy levels by 1 to 2 months postpartum and remained at those levels at least until 12 months postpartum. Decreases in mood and physical quality of life may be natural occurrences as a result of the physical and physiological changes that occur during pregnancy. Higher overall gestational weight gain was associated with worsened mood and lower physical quality of life from early to late pregnancy, so it is essential to include emotional and well-being aspects and support in lifestyle interventions and clinical counseling during pregnancy to aid in limiting excess gestational weight gain and improve future health in both women and their infants.

## Additional file

Additional file 1: SmartMoms® Intervention Lessons. Intervention lesson topics and delivery outline included in the SmartMoms® Intervention In-Person and Phone groups. An example. Lesson titled *Motivation and Goal Setting* was included to demonstrate the layout of SmartMoms® Intervention lesson topics. (DOCX 216 kb)

## Abbreviations

BDI-II: Beck Depression Inventory II
BMI: Body mass index
GWG: Gestational weight gain
IOM: Institute of Medicine
LIFE-Moms: Lifestyle Interventions for Expectant Moms Consortium
MCS: Mental health composite score
PCS: Physical health composite score
Ppm: Postpartum
Preg: Pregnancy
SD: Standard deviation
SE: Standard error
SF-12: Rand 12-Item Short Form
