# Supplementary material for: Mood and quality of life changes in pregnancy and postpartum and the effect of a behavioral intervention targeting excess gestational weight gain in women with overweight and obesity: a parallel-arm randomized controlled pilot trial
Source: BMC Pregnancy Childbirth. 2019 Jan 29;19:50. doi: 10.1186/s12884-019-2196-8 (PMC6352352; doi:10.1186/s12884-019-2196-8)
Supplement: Supplementary file 1 — SmartMoms® Intervention Lessons. Intervention lesson topics and delivery outline included in the SmartMoms® Intervention In-Person and Phone groups. An example. Lesson titled Motivation and Goal Setting was included to demonstrate the layout of SmartMoms® Intervention lesson topics. (DOCX 216 kb) [file 12884_2019_2196_MOESM1_ESM.docx]

**Additional File 1.**

**SmartMoms^®^ Intervention Lessons**

Intervention Outline:

**Lessons**

**SmartMoms-In-Person**

**SmartMoms-Phone**

Module A

*Weight Gain During Pregnancy & Personalized Calorie and Exercise Goals**

*Individual*

*Individual***

Motivation and Goal Setting

Group

Phone

Vitamins and Minerals

Group

Phone

Stimulus Control: Incorporating Mindfulness into Your Environment

Group

Phone

Eating Patterns and Building Social Support

Individual

Phone

High Fiber Eating and Carbohydrate Intake

Group

Phone

Importance of Physical Activity During Pregnancy

Group

Phone

Dietary Fats and Fluid Intake

Group

Phone

Behavior Chains: How Our Eating and Exercise Behaviors Can Be Managed

Individual

Phone

Management of Food Cravings and Snacking

Group

Phone

Managing Time and Stress and the Importance of Sleep

Group

Phone

Coping with Overeating and Hunger Management (Portion Control Training)

Group

Phone

Module B

How to Overcome Barriers to Success

Individual

Phone

Dietary Protein and Progress Review

Group

Phone

Ways to Increase Activity and Walking

Individual

Phone

Mindfulness and Relaxation Techniques

Group

Phone

The Importance of Sleep and Time Management

Group

Phone

*Progress Review and Future Goals**

*Individual*

*Individual***

*The study relies on open enrollment; when participants enroll they receive the lesson that is being delivered that week. The

exception is the first and last session with the counselor, where the participant receives her personal goals and follow-up plan.

The lesson sequence in this table is a sample sequence for an individual participant.

**For SmartMoms-Phone participants, the first and last sessions are in person and will include delivery and return of equipment. All

other lessons are delivered via Smartphone.

For each intervention lesson, the structure is completed as below but with the topic and goals of interest:

1. Need to know information first
2. Reiteration of the most important aspect of the material at the end
3. Goals for the upcoming week

Example Lesson: Motivation and Goal Setting Lesson

**Motivation and Goal Setting**

**Introduction to Motivation**

Making changes to your eating habits in order manage gestational weight gain for you and your baby, and keep your weight in the recommended “zone” takes work and motivation. Maintaining your motivation is **key** to managing body weight now and in the future. In this session, you will learn to evaluate your specific motivation and develop strategies to maintain your motivation as you go through this process.

**What is Your Motivation to Manage Gestational Weight Gain and Adhere to the Recommended Weight Graph?**

Patricipants in this study generally have one key motivating factor: to manage their gestational weight gain for the health of themselves and their baby. Women whose weight gain during pregnancy is outside of the recommended ranges are at increased risk for complications such as:

- pregnancy-associated hypertension
- gestational diabetes complications during labor and delivery
- postpartum weight retention and obesity

The chances of successfully breast feeding are also lowered with excessive maternal weight gain. Women whose gestational weight gain is outside of the recommended ranges also put their baby at increased risk of developing obesity-related consequences such as insulin-resistant diabetes.

Your motivation for keeping your weight within the recommended zone may change over the course of your prengancy (and the study), and different challenges may arise over time. Discussing these changes with your counselor and in the Expecting Success group setting will help you stay motivated to keep your weight in recommended weight zone.

**Stages of Motivational Readiness for Behavior Change**

There is a theory known as the “Stages of Motivational Readiness for Behavior Change” that may help you to understand what the underlying motivational factor for your desire to manage gestational weight gain.

This theory basically suggests that you move through various stages as you focus on changing your eating behaviors. As you move through these stages, the factors that motivate you to stay on course can actually change depending on the stage that you are in.

The FIVE stages include:

- Precontemplation
- Contemplation
- Preparation
- Action
- Maintenance

These stages are illustrated below.

**Precontemplation**


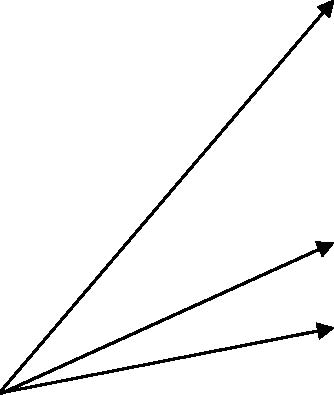


**Contemplation**

**Preparation**

**Action**

**Lapse/ Relapse**

**Maintenance**

Now, let’s examine how you have moved through this model as you decided to manage your gestational weight gain.

- **Precontemplation Stage**

At some point, you were in this stage. At this point you were not thinking about making any changes to your eating habits during pregnancy.

*-When were you in this stage?*

- **Contemplation Stage**

Then, something triggered a move to this stage. At this point you wanted to begin to do something to manage your gestational weight gain, but you were not quite sure what you would do. From a motivational perspective, it is important for you to think about why you moved into the contemplation stage.

*-What was it that triggered you? What motivated you in wanting to enroll in the Expecting Success program and begin to make some changes?*

- **Preparation Stage**

Next you were able to shift into this stage***.*** At this point you decided to enroll in the Expecting Success Program.

*-Why did you make the decision to enroll in the Expecting Success Program?*

- **Action Stage**

Then you moved into this stage—which is where you are right now, engaging in a behavior change program to assist you with managing your gestational weight gain for the health of you and your baby.

*-What caused you to move into this stage?*

- **Maintenance Stage**

After you have been successful at adhering to the weight graph for a few weeks or months, you will move into a maintenance stage. During this time you will be monitoring your weight as it fluctuates within the zone to maintain the optimal health of you and your baby. You should be aware that during this time people may have a lapse, take care that it doesn’t become a relapse; depend on your counselor, your support people, and your own motivation to stay in the zone for the health of you and your baby!.

So, let’s focus on what got you to this point – the point where you decided to take action.

**Understanding the Role Motivation Plays on Success**

**Planning to Maintain Motivation**

A good plan includes several strategies to maintain motivation. Here are some common strategies to maintain motivation:

- Setting specific short-term and long-term goals
- Inviting support from others
- Keep records of your progress
- Focus on positive thinking
- Remembering your purpose and the health of you and your baby

What are sometimes overlooked are the small, less obvious factors. You may begin to feel more energetic in the second trimester, which may increase your motivation to keep your weight gain within zone.

Now is a great time to make records of why you want to manage gestational weight gain throughout the second and third trimesters. As pregnancy progresses, these records can serve as reminders and motivation for times when you need that extra boost.

When making a record, keep in mind:

- *Why were you motivated to meet your weight goal in the first place?*
- *What helps you stay motivated?*
- *What makes it hard for you to stay motivated?*
- *What you can do to overcome these barriers to motivation?*

**Goal Setting**

The goal of this section is to identify your specific gestational weight gain goals. You have been provided with a calorie target of how much your calories should increase each trimester. You were also given an individualized weight graph which depicts the rate of weight gain that is healthy for you and your baby. Your main goal is to keep your weight within this “zone” over the course of the second and third trimesters. We will help you learn to set small goals along the way that will help you achieve the overall goal of the study.

Goals are more easily met if you have a detailed, specific plan to follow.

A good plan identifies:

- The behaviors/goals to be accomplished
- How it will happen
- When it will happen

**SMART Goal Setting**

**S**pecific: All goals should have a specific behavior that is targeted and precisely define the behavior that is to be accomplished.

Behavior-Specific – I will eat an additional 175 calories/day as prescribed to me 12/14 days so that I can stay in the zone.

Outcome-Specific – I will gain 2 pounds in the next two weeks, staying within my weight zone.

**M**easurable: All goals should be measurable in order to determine if the goal was in fact accomplished.

**A**ttainable: Goals should be challenging yet realistic enough that they can be achieved without perfection.

**R**eward: (Purpose) Oftentimes goals are set without a clear reason as to why they are important. By identifying and reminding yourself why the goal is important, you are more likely to stay committed to achieving it.

In addition, giving yourself a non-food external reward (buying a new CD, getting a manicure, etc.) or an internal reward (praising yourself) can be motivating.

**T**ime frame: Having an end date for your goals allows you to assess your progress and set new goals that are important to you. Having an end date also allows you to modify a goal if you were unable to accomplish it in the designated time frame. Usually short term goals are assessed after 1-2 weeks to make sure they are moving you toward your long term goals.

**Change the following goals so they meet the SMART principle.**

“I am going to get back on track with my eating and gain weight at a healthy rate.”

SMART goal: __________________________________________________________

“Over the next few weeks I will stay in the weight zone (or work on getting back in the zone).”

SMART goal: __________________________________________________________


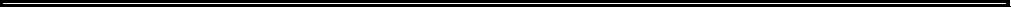


| **Helpful goals are**: | **Example** |
| --- | --- |
| Positive | I will plan 500 calorie dinner menus.  ***Instead of negative***: I will stop eating so much. |
| Specific | I will take and eat a Healthy Choice meal for lunch.  ***Instead of vague***: I will grab something at work for lunch. |
| Something under your control | I will stop buying cartons of ice cream and ask my husband to only eat ice cream when he eats out.  ***Instead of what you can’t control***: I will get my husband to stop eating ice cream. |
| Time Limited | I will be manage my weight gain be in the recommended weight zone prescribed to me by October 12.  ***Instead of open-ended***: I will manage my weight. |
| Small enough so you can reach them | I will eat out 2 times per month.  ***Instead of***: I will never eat out again. |
| Broken into small steps | I will buy carrots and celery at the grocery store, cut them into sticks, and put them in the refrigerator in small plastic  ***Instead of not broken down***: I will eat carrots and celery sticks for lunch. |
| Related to a reward | I will buy a copy of my favorite magazine if I pack my l lunch 3 times this week.  ***Instead of:*** I pack my lunch 3 times this week. |

| **Take this not-so-helpful goal** | **Make it:** | **Example of a more helpful goal** |
| --- | --- | --- |
| I will stop eating dessert. | Positive |  |
| I will eat less bread. | Specific |  |
| My kids will stop eating cookies for snack. | Under your control |  |
| My boss will stop bringing doughnuts in for everyone on Friday mornings. | Under your control |  |
| I will eat salads with low-calorie dressing. | Time specific |  |
| I will never eat ice cream again. | Small enough |  |
| I will go to restaurants that have more low-calorie choices on the menu. | Broken into small steps |  |
| I will eat fruit for a bedtime snack tonight. | Related to a reward |  |

**Session Activity: Setting SMART Goals**

***The discipline you learn and character you build from setting and achieving a goal can be more valuable than the achievement of the goal itself.***

**-Bo Bennett**

**Set your own SMART goal:**

I will (*be specific, make it measurable and attainable*):

________________________________________________________________________________________________________________________________________________________________________________________________

I will complete this by (date): ______________________________________

If I am going to accomplish this, it will be important for me to also do the following *(include dates where applicable)*:

____________________________________________________________________________________________________________________________________________________________________________________

If I encounter difficulties in accomplishing this goal I will:

________________________________________________________________________________________________________________________________________________________________________________________________

If I accomplish this goal, I will reward myself by:

________________________________________________________________________________________________________________________________________________________________________________________________


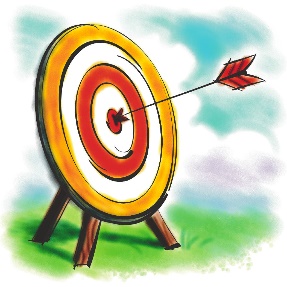


**End of Session: Next Steps**

**To Do This Week**

- Keep track of food intake daily with SmartPhone or food records.
- Take prenatal vitamin daily.
- Wear your activity monitor daily and track daily steps.
- Make a record of what is motivating you to manage your weight gain.
- Complete the SMART goal worksheet to set at least one goal for yourself over the next week.
- At the end of the week, evaluate your efforts to make this change.
- Come to the next group session on: ________________________________
- Other:_________________________________________________________
